# Driving: A Road to Unhealthy Lifestyles and Poor Health Outcomes

**DOI:** 10.1371/journal.pone.0094602

**Published:** 2014-06-09

**Authors:** Ding Ding, Klaus Gebel, Philayrath Phongsavan, Adrian E. Bauman, Dafna Merom

**Affiliations:** 1 Prevention Research Collaboration, Sydney School of Public Health, University of Sydney, Sydney, New South Wales, Australia; 2 School of Public Health, Tropical Medicine, and Rehabilitation Sciences, James Cook University, Cairns, Queensland, Australia; 3 School of Science and Health, University of Western Sydney, Penrith, New South Wales, Australia; Arizona State University, United States of America

## Abstract

**Background:**

Driving is a common part of modern society, but its potential effects on health are not well understood.

**Purpose:**

The present cross-sectional study (n = 37,570) examined the associations of driving time with a series of health behaviors and outcomes in a large population sample of middle-aged and older adults using data from the Social, Economic, and Environmental Factor Study conducted in New South Wales, Australia, in 2010.

**Methods:**

Multiple logistic regression was used in 2013 to examine the associations of usual daily driving time with health-related behaviors (smoking, alcohol use, diet, physical activity, sedentary behavior, sleep) and outcomes (obesity, general health, quality of life, psychological distress, time stress, social functioning), adjusted for socio-demographic characteristics.

**Results:**

Findings suggested that longer driving time was associated with higher odds for smoking, insufficient physical activity, short sleep, obesity, and worse physical and mental health. The associations consistently showed a dose-response pattern and more than 120 minutes of driving per day had the strongest and most consistent associations with the majority of outcomes.

**Conclusion:**

This study highlights driving as a potential lifestyle risk factor for public health. More population-level multidisciplinary research is needed to understand the mechanism of how driving affects health.

## Introduction

Spending large amounts of time driving to work and other destinations is a common part of modern society [1]. However, it was not until the recent years that cars were identified as an unsustainable mode of travel, with the primary concerns being their detrimental impact on the environment, road injuries, and reduced opportunities for active transport or safe outdoor play [2].

More recently, the research focus has shifted to the impact of driving as a health-related behavior. Several studies have shown that lengthy car commuting perpetuates conditions that compromise individuals' health, which includes stress caused by traffic congestion, searching for parking, interacting with other drivers and safety concerns; phenomena characterized as “travel impedance” [3], [4]. Additionally, prolonged commuting replaces time devoted to other behaviors which may affect health, resulting in insufficient sleep [5], [6], reduced time spent with family members or friends [2], [4], insufficient leisure time physical activity or time for preparation of food [3], [7]. Last, transport-related sitting is one domain of sedentary behavior, which has been linked to increased chronic disease risks [8]. An American cross-sectional study found that each hour in the car was associated with a 6% increase in the odds of obesity [9].

Driving is ubiquitous worldwide; however, the understanding of its health implications is inadequate. Most preliminary research has been limited to the context of commuting to and from work, which includes driving, but also other modes of transportation, such as public transport [3], [10], [11]. It is therefore not clear whether those adverse health outcomes are unique to long-distance commuters or to driving in general, regardless of purposes. Furthermore, despite the fact that health effects of driving are likely to be multi-dimensional, most studies to date focused on one or a small number of health outcomes. The present study aimed to explore the associations of driving time for any purposes with a series of health behaviors and outcomes in a large population-sample of middle-aged and older Australians. The hypothesis is that longer exposure to driving is associated with worse health behaviors and outcomes.

## Methods

### Sampling and Procedures

This cross-sectional analysis was based on the Social, Economic, and Environmental Factor (SEEF) Study, which was conducted as a follow-up sub-study of the SAX Institute's 45 and Up Study [12], a large cohort study of adult residents aged 45 years and older in New South Wales, Australia (2006–2008; n = 267,153). Participants were randomly sampled from the Medicare Australia database, which includes all residents in the state of New South Wales. In 2010, the first 100,000 participants of 45 and Up were invited by mail and 60,404 participated in SEEF by completing the consent form and the questionnaire and mailed them back in prepaid envelopes to the study coordinating center (response rate of 60.4%). The 45 and Up Study was granted ethical approval by the University of New South Wales Human Research Ethics Committee (HREC 05035/HREC 10186) and the SEEF Study by the University of Sydney Human Research Ethics Committee (ref no. 10-2009/12187).

### Measurement

Driving was measured using a single-item question “About how many hours in each 24 hour day do you usually spend driving?”

### Health Behaviors

Smoking risk was defined as being a current regular smoker. Alcohol risk was defined as consuming more than 14 servings of alcohol per week. Dietary risk was defined as not meeting the Australian dietary guidelines for fruit and vegetable intake (2 servings of fruit + 5 servings of vegetables per day) [13]. Physical activity risk was defined as engaging in less than 150 minutes/week of moderate-to-vigorous intensity activity based on the Active Australia Survey [14]. Excessive sitting was defined as 8 hours or more of sitting in a 24 hour-day based on findings from 45 and Up [15]. Insufficient sleep was defined as sleeping less than 7 hours/day based on a meta-analysis on sleep duration and mortality [16].

### Health Outcomes

Obesity was measured as body mass index (BMI)≥30 based on self-reported height and weight. Previous research among a random sub-sample of 45 and Up showed excellent agreement between BMI categories from self-reported and measured data [17]. Overall self-rated health was measured using a single-item question (“In general, how do you rate your overall health?”) from the Medical Outcomes Study 12-Item Short-Form Health Survey (SF-12). Quality of life was assessed using a parallel question “In general, how do you rate your quality of life?” Psychological distress was measured using Kessler-10, which has been validated in the Australian population [18]. Respondents' scores of 22 and above were coded as “high/very high psychological distress”, which correlates with a clinical diagnosis of depression and anxiety disorders needing interventions [18], [19]. Time stress was measured using a question previously used by the Australian Bureau of Statistics [20] “How often do you feel rushed or pressed for time” and those who answered “always” or “often” were coded as “stressed for time.” Social functioning was measured using one item from SF-12 “How much time during the past four weeks have your physical health or emotional problems interfered with your social activities?” Those who reported “all/most of the time” were coded as “poor social functioning.”

### Data Analysis

Analyses were restricted to those with complete data, aged 75 and below, and who reported any driving (n = 36,430∼37,570 for different outcomes). Descriptive analyses of all variables were conducted to compare across participants with different amounts of driving using chi-square tests and ANOVA. Logistic regression analysis was conducted for each health behavior/outcome variable adjusted for age, sex, marital status, employment status, educational attainment, the number of motor vehicles per person in the household, rural/urban residence, and area-level socioeconomic disadvantage (measured by the Index of Relative Socio-Economic Disadvantage). Sex by driving and education by driving interactions were tested in all models for potential effect modification.

## Results

The demographic characteristics of the study sample are presented in Table 1. Overall, the sample (n = 37,570) averaged 61 years of age, 54% were females, 81% were married, 58% were in paid employment, and less than 30% had a university degree. The average driving time was 84 minutes/day (SD = 82) and the median was 60 minutes/day. Those who drove more were more likely to be younger, male, with lower educational attainment, in paid employment, and had more motor vehicles per person in the household.

**Table 1:** Demographic, behavioral, and health characteristics by usual daily driving time of adults aged 47–75 in New South Wales, Australia, 2010 (n = 37,570).

	<30 min	31–60 min	61–120 min	>120 min	Overall
**Demographics**					
Age: mean years (SD)	62.20 (7.05)	61.33 (7.10)	60.13 (7.02)	59.85 (6.66)	61.15(7.08)
Female (%)	63.88	55.64	49.57	35.55	54.34
Married/de-facto (%)	80.52	81.33	78.93	80.46	80.65
Education (%)					
Up to 10 years	25.90	26.41	24.88	31.70	26.50
High school/Diploma	41.58	43.31	44.62	49.73	43.79
University	32.52	30.28	30.50	18.57	29.72
In paid employment (%)	47.36	53.84	68.82	79.13	57.58
No. of motor vehicles per person: mean (SD)	0.89 (0.36)	0.93 (0.40)	0.95 (0.46)	1.02 (0.55)	0.93 (0.42)
Rural residence (%)	36.40	30.33	25.37	30.74	30.64
**Health behaviors**					
Smoking regularly	3.65	5.00	5.49	7.27	5.03
Alcohol > 2 servings/day	13.21	15.37	15.14	18.34	15.17
Not meeting the dietary recommendation for fruits and vegetables	73.49	76.51	78.08	79.16	76.45
Insufficiently active (<150 min/week)	16.01	17.55	20.21	24.72	18.38
Excessive sitting (≥8 hr/day)	17.62	18.93	21.34	20.87	19.29
Insufficient sleep (<7 hr/day)	13.99	15.25	19.22	24.03	16.51
**Health outcomes**					
Obesity	19.47	23.49	25.53	29.91	23.64
Poor/fair self-rated health	10.62	11.01	10.24	12.05	10.89
Poor/fair quality of life	6.26	6.32	6.89	8.71	6.62
High psychological distress	5.68	5.67	6.35	7.34	5.94
Stressed for time	25.66	27.04	34.69	35.85	28.96
Health interfering with social activities	15.15	15.92	16.29	17.25	15.96

CI = confidence interval; hr = hours; min = minutes.

All tests for trend were significant at *p*<0.001 except for marital status (*p* = 0.057) and self-rated health (*p* = 0.288).

### Driving and Health Behaviors

Based on unadjusted descriptive statistics (Table 1), there was a consistent trend that higher prevalence rates of smoking, excessive alcohol use, insufficient fruit and vegetable consumption, physical inactivity, and insufficient sleep were observed among those with higher driving time. When adjusted for socio-demographic characteristics, there was a positive linear trend for smoking, physical inactivity, and insufficient sleep across dose categories of driving (*p*<0.001), and for these three health behavior outcomes statistically significant associations occurred from the level of 31-60 min/day of driving (Table 2). Surprisingly, those who reported driving for more than 120 min/day were less likely to sit for more than 8 hours per day (OR = 0.88, *p* = 0.03) and there was an overall inverse association between driving time and excessive sitting (*p* = 0.042). No driving time by sex/education interaction was significant at *p*<0.05.

**Table 2:** Adjusted odds ratios^a^ for the associations between daily driving time and health behaviors and outcomes among driving adults aged 47–75 in New South Wales, Australia, 2010.

		31–60 min	95% CI	61–120 min	95% CI	121 min+	95% CI	*P* for trend
**Health behaviors**	**n**							
Smoking regularly	37,563	1.36***	1.18, 1.56	1.42***	1.21, 1.68	1.73***	1.43, 2.09	<0.001
Alcohol >2 servings/day	37,030	1.04	0.96, 1.13	0.93	0.84, 1.03	0.96	0.85, 1.08	0.141
Not meeting the dietary recommendation for fruits and vegetables	37,563	1.05	0.99, 1.12	1.04	0.96, 1.13	0.96	0.87, 1.07	0.506
Insufficiently active (<150 min/week)	37,563	1.10**	1.02, 1.19	1.28***	1.17, 1.40	1.57***	1.41, 1.74	<0.001
Excessive sitting (≥8 hr/day)	36,877	0.99	0.92, 1.06	0.94	0.86, 1.03	0.88*	0.79, 0.99	0.042
Insufficient sleep	37,452	1.10*	1.02, 1.19	1.41***	1.28, 1.54	1.86***	1.67, 2.07	<0.001
**Health outcomes**								
Obesity	35,183	1.30 ***	1.21, 1.40	1.50***	1.38, 1.63	1.78***	1.61, 1.97	<0.001
Poor/fair self-rated health	37,468	1.06	0.97, 1.16	1.08	0.96, 1.21	1.29***	1.12, 1.48	<0.001
Poor/fair quality of life	36,423	0.99	0.89, 1.11	1.15	1.00, 1.32	1.43***	1.21, 1.70	<0.001
High/very high psychological distress	37,191	0.98	0.87, 1.10	1.13	0.98, 1.31	1.33**	1.11, 1.58	<0.001
Stressed for time	37,309	1.01	0.95, 1.08	1.28***	1.19, 1.39	1.42***	1.29, 1.57	<0.001
Health interfering with social activities	37,366	1.08	1.00, 1.16	1.15**	1.05, 1.27	1.23***	1.10, 1.39	<0.001

Reference category: 1–30 min driving/day.

CI = confidence interval; hr = hours; min = minutes.

*P<0.05, **P<0.01, ***P<0.001 when comparing with the reference category (1–30 min/day of driving).

Adjusted for age, sex, marital status, employment status, educational attainment, and number of motor vehicles per person in the household, rural/urban residence, and Socio-Economic Indexes for Areas disadvantage scores.

### Driving and Health Outcomes

Based on unadjusted analyses, those who spent more time driving were more likely to report obesity, fair/poor quality of life, high/very high psychological distress, time stress, and having physical health or emotional problems that interfered with social functioning (Table 1). When adjusted for socio-demographic characteristics, there was a positive linear trend for all health outcomes across dose categories of driving (*p*<0.001) and the significance occurred from 31–60 min/day for obesity, from 61–120 min/day for time stress and social functioning, and at more than 120 min/day for self-rated health, quality of life, and psychological distress (Table 2).

For all health outcomes, there was no significant effect modification by sex or educational attainment at *p*<0.05. No driving time by sex/education interaction was significant at *p*<0.05.

## Discussion

The current study is amongst the first to examine the associations of driving time for any purpose with a range of health behaviors and outcomes. Results confirmed the hypothesis that driving is associated with different aspects of physical and mental health and health behaviors when socio-demographic differences are adjusted for. While most relationships showed a dose-response pattern, 120 minutes and more per day of driving had the strongest and most consistent associations with the majority of outcomes. This indicates that more than 2 hours of driving per day, which accounts for at least 12% of one's waking hours, is particularly detrimental to the health of middle-aged and older adults. While a previous study linked driving time to an alleviated risk for cardiovascular disease (CVD) mortality [21], the mechanism for this association is largely unclear. Results from the present study could help delineate the observed association between driving and CVD mortality.

### Driving and Health Behaviors

Our findings highlight the clustering of poor health-related behaviors with driving. Associations with insufficient amount of sleep, physical inactivity, and regular smoking were significant and showed clear dose-response patterns. While other studies have highlighted the association of long commutes with sleep deprivation [5], [10] and physical inactivity [5], to our knowledge this is the first study to demonstrate that long driving time in general is associated with smoking. Time trade-offs are likely to be the most plausible explanation for compromised sleep and lack of physical activity, as an American time-use study indicated that more than 20% of the time of a 120-minute commute was taken from physical activity [5]. The mechanism by which driving induced smoking is less clear; it could be either a reaction to driving-induced stress, idle “time to kill”, or the “coupling” of behavioral rituals (e.g., driving) and the sensory/physiological reinforcements of smoking through second-order conditioning [22]. In this case, it is possible that each episode of entering the car “for a drive” or “sitting and driving” becomes a cue that evokes strong craving for smoking [23].

The lack of association with alcohol is not surprising given the risk associated with driving under the influence of alcohol. The lack of association with dietary behavior should be interpreted with caution because the measures only concerned fruit and vegetable intake, not other food items such as coffee, sweets, and sugar flavored beverages. Future studies should particularly include measures regarding fast food intake, dining out, and the consumption of energy-dense foods as previous studies have demonstrated that long commutes were associated with less time spent in food preparation [5], [7]. The slightly inverse association of driving time with excessive sitting is unexpected, as driving (or sitting for transport) contributes to overall sitting. This may be explained by other domains of sedentary behavior, such as occupational and recreational sitting, which may be disproportionately more common among those who spend less time driving. Regardless, our finding also implies that it may be oversimplified to assume that driving harms health mainly through prolonged sitting [23].

### Driving and Health Outcomes

This study found a strong relationship between driving time and self-reported health and well-being, such as poor self-rated health, poor quality of life and high/very high psychological distress. The mechanisms that may moderate or mediate the associations between driving and mental health outcomes are likely to be multidimensional and complex. The associations between driving and health may be mediated by “travel impedance” [24], [25], such as exposure to stressful driving conditions, including adverse road environments, dealing with traffic congestion causing delays, and behaviors of other road users. Consequently, one would expect that the mental workload required for driving would have some measurable impact on health and well-being. Another possible mechanism relates to physical and leisure-time activities. Given the well-established relationship between physical activity and physical and mental health [26], it is plausible that reduced time for health-related activities as a result of more time spent driving will have consequences on health and well-being. The trade-off between driving and spending time with one's social network [27] is another important factor since limited social interactions have been shown to adversely impact health outcomes, such as mental health and mortality [28], [29]. Having supportive social interactions may provide a buffer against stressful driving experiences. Empirical work would be necessary to confirm these possible mechanisms.

In this study, the strength of the associations of driving with time stress and health-related social functioning are substantial, observed from an hour and more of driving per day. Whether time pressure leads to more driving or spending more time driving increases the odds of feeling stressed for time, the finding highlights the potential role of time stress in the relationship between driving time, health, and well-being. Finally, the link between driving time and obesity observed in this study is consistent with previous studies [9], [30], [31] and reaffirms driving as a potentially important risk factor for cardio-metabolic health.

### Strengths and limitations

Key strengths of the study included a large population-based sample and the assessment of comprehensive behavioral and health outcomes using validated measures. The consistent patterns of associations across health behaviors and health outcomes enhance the validity of findings. The question about driving focuses exclusively on driving, rather than commuting, which not only includes other modes of transportation (i.e, train, bus, taxi) but also precludes driving for other purposes such as doing errands, chauffeuring children, and driving for recreational purposes. This is important because in Australia work commutes only accounted for 27% of the total kilometers traveled by passenger vehicles [32], suggesting that a large proportion of driving time is not accounted for in commuting based research. However, such a comprehensive measure has its merits, but also limitations, depending on the potential underlying mechanism through which driving affects health. For example, driving impedance is relevant to driving for all purposes while the lack of social interactions may be more relevant to commuting and less so to chauffeuring children or recreational driving. A cross-sectional study design is a limitation of the study. Although it is less plausible that unhealthy lifestyles are antecedents to driving behavior, it is possible that those with poor health depend more on driving because they have limited physical capacity for active travel (e.g., walking or cycling). Typical for large population-based studies, another limitation is that all measures were based on self-report data, which are subject to biases. The study could also be improved by including more detailed measures about driving, such as the duration of each trip (bout), to pinpoint risk associated with one episode of prolonged driving or cumulative driving time over multiple trips. Furthermore, future studies should include other modes of transport, such as public transportation, so that one can isolate effects of driving from overall daily travel time.

## Conclusion

Driving is a potential risk factor for a cluster of health behaviors and outcomes among middle-aged to older adults. To better understand the mechanisms for the health risk of driving, future transportation and time use surveillance surveys should incorporate health-related questions to provide opportunities for testing more specific hypotheses about transportation and health. Although further empirical evidence is needed, future lifestyle interventions and transportation planning initiatives may consider reducing driving time as a potential strategy for promoting health and well-being.
